# Impacts of Micro/Nanoplastics Combined with Graphene Oxide on *Lactuca sativa* Seeds: Insights into Seedling Growth, Oxidative Stress, and Antioxidant Gene Expression

**DOI:** 10.3390/plants13243466

**Published:** 2024-12-11

**Authors:** Xuancheng Yuan, Fan Zhang, Zhuang Wang

**Affiliations:** 1 Jiangsu Key Laboratory of Atmospheric Environment Monitoring and Pollution Control, Collaborative Innovation Center of Atmospheric Environment and Equipment Technology, School of Environmental Science and Engineering, Nanjing University of Information Science and Technology, Nanjing 210044, China; 202212480158@nuist.edu.cn; 2 College of Environmental Science and Engineering, Yangzhou University, Yangzhou 225127, China

**Keywords:** microplastics, nanoplastics, phytotoxicity, mixture toxicity

## Abstract

Global pollution caused by micro/nanoplastics (M/NPs) is threatening agro-ecosystems, compromising food security and human health. Also, the increasing use of graphene-family nanomaterials (GFNs) in agricultural products has led to their widespread presence in agricultural systems. However, there is a large gap in the literature on the combined effects of MNPs and GFNs on agricultural plants. This study was conducted to explore the individual and combined impacts of polystyrene microplastics (PSMPs, 1 μm) or nanoplastics (PSNPs, 50–100 nm), along with agriculturally relevant graphene oxide (GO), on the seed germination and seedling growth of lettuce (*Lactuca sativa*). The results showed that the combined effects of mixtures of PSMPs/PSNPs and GO exhibited both synergism and antagonism, depending on different toxicity indicators. The cellular mechanism underlying the combined effects on the roots and shoots of seedlings involved oxidative stress. Three SOD family genes, namely, Cu/Zn-SOD, Fe-SOD, and Mn-SOD, played an important role in regulating the antioxidant defense system of seedlings. The extent of their contribution to this regulation was associated with both the distinct plastic particle sizes and the specific tissue locations within the seedlings.

## 1. Introduction

Micro/nanoplastics (M/NPs) have been detected in the global environment, and their contamination has garnered widespread attention from both the scientific community and the public [1,2]. Moreover, M/NPs in food are gradually becoming recognized by regulatory authorities and the public [3]. The physicochemical properties of M/NPs, including their small particle size, high specific surface area, hydrophobicity, and resistance to degradation, have also raised concerns regarding the potential human health and ecological risks associated with them [4,5]. Given the close relationship between crop plants and human life and the direct impact M/NPs can have on the growth and development of these plants, there is a heightened need for research on the effects of M/NPs on crop plants [6].

In recent years, nanotechnology has gained significant attention and application in agriculture and food [7,8]. Specifically, graphene-family nanomaterials (GFNs) with diverse structural and chemical properties are increasingly used in the fields of agriculture [9,10,11]. With the growing use of GFNs, there are concurrent public concerns regarding their safety upon introduction into the agricultural ecosystem [12,13]. GFNs can exert an impact on plant growth spanning from morphological to biochemical scales, encompassing effects on root growth, membrane structure disruption, antioxidant system alteration, and hormone concentration changes, which can ultimately be traced to modifications at the gene expression level [13]. In addition, the release of GFNs into the environment can enhance biological exposure in various ways [14]. The widespread presence of M/NPs and GFNs may lead to their coexistence in the agricultural environment, potentially causing joint impacts on crop species. Notably, the unique physicochemical characteristics of M/NPs and GFNs, such as large specific surface area and hydrophobicity, can result in “Trojan horse” effects, affecting their transport and toxicity in the environment [15,16]. However, research on the combined effects of M/NPs and GFNs is still limited compared to studies on their individual effects on agricultural plants.

In this study, we investigated the individual and combined effects of polystyrene microplastics (PSMPs; 1 μm) and nanoplastics (PSNPs; 50–100 nm) along with agriculturally relevant graphene oxide (GO) on the seed germination and early seedling growth of lettuce (*Lactuca sativa* L. var. *Ramose* Hort). Lettuce is a crucial and widely consumed vegetable worldwide [17]. Seed germination and seedling growth are critical stages in the plant growth cycle [18]. The primary objectives of this research were to (i) examine the separate and combined impacts of PSMPs/PSNPs and GO on lettuce seed germination and seedling growth, (ii) elucidate the separate and combined impacts of PSMPs/PSNPs and GO on different tissues of lettuce seedlings at the cellular and genomic levels, (iii) evaluate the interactions of PSMPs/PSNPs with GO from the epigenetic level to the genomic levels, (iv) establish a correlation between oxidative stress effects and epigenetic growth effects, and (v) identify the contribution of different types of antioxidant-related genes in regulating antioxidant activity.

## 2. Materials and Methods

### 2.1. Test Materials

The selected PSMPs and PSNPs were purchased from Nanjing Xianfeng Nanomaterials Technology Co., LTD. (Nanjing, China) and Aladdin Industrial Co., LTD. (Shanghai, China), with primary sizes of 1 μm and 50–100 nm, respectively. GO with a primary particle size of 0.5–3 μm and a thickness of 0.55–1.2 nm (specific surface area of 100–400 m^2^/g, purity greater than 99%) was purchased in powder form from Aladdin Industrial Co., LTD. (Shanghai, China). A suspension of GO was prepared by dispersing the powder in sterile, deionized (DI) water, which was magnetically stirred for one hour to ensure that GO was properly dispersed before further use. The reserve suspension was then stored at 4 °C until ready for use. The seeds of lettuce (*L. sativa*) were provided by the Institute of Vegetables and Flowers of the Chinese Academy of Agricultural Sciences.

### 2.2. Physicochemical Analysis

The morphology of PSMPs/PSNPs and GO alone and in combination was characterized in the DI water using transmission electron microscopy (TEM, JOEL 2100f, JOEL Ltd., Tokyo, Japan). Size distributions and hydrodynamic diameters of particles suspended in the DI water were analyzed using a ZetaSizer (Nano ZS90, Malvern Instruments Ltd., Worcestershire, UK).

### 2.3. Germination of Lettuce Seeds

Neatly developed and full lettuce seeds were selected and sterilized using sodium hypochlorite (0.5% *w*/*v*) for a duration of 15 min. Subsequently, the sterilized seeds were rinsed three times with DI water. Twenty-five seeds were randomly selected from the sterilized batch and placed in a glass Petri dish, which was then covered with qualitative filter paper, serving as the treatment. The lettuce seeds were exposed to varying concentrations of 0 (control), 0.1, 1, 10, and 100 mg/L of isolated PSMPs, PSNPs, and GO. The concentrations of the binary mixtures of PSMPs/PSNPs and GO were set by combining the individual concentrations of the components in pairs. Subsequently, each experimental group was sealed and placed within an artificial climate chamber, where they were cultivated for 7 d under controlled conditions of 18 ± 2 °C and 60% humidity in complete darkness. Furthermore, the experiment was replicated twice, with each iteration conducted at an interval of seven days. Germination potential and germination rate were recorded after 3 and 7 d, respectively. Germination rate (%) = (number of germinated seeds/total number of tested seeds) × 100%. Over 7 d, the root elongation, shoot length, and total fresh weight of lettuce were measured.

### 2.4. Antioxidant Activity Assays

Total protein (TP) content, superoxide dismutase (SOD) activity, malondialdehyde (MDA) content, and glutathione (GSH) content in the roots and shoots of lettuce seedlings (7 d) were determined using commercial kits purchased from Nanjing Institute of Jiancheng Biological Engineering (Nanjing, China). To determine antioxidant activity, the roots and shoots of lettuce seedlings were cut, separated, and subsequently ground using traditional mortar and pestle on ice. Then, 3 mL of phosphate buffer salt solution was added. The ground sample was then centrifuged at 1000× *g* at 4 °C for 10 min. The collected supernatant was used to measure the TP content, SOD activity, MDA content, and GSH content. The procedure for the determination of biomarkers followed the instructions of the kit manufacturer. Each experimental group comprised three parallel replicates.

### 2.5. Antioxidant Gene Expression Assays

The lettuce seedlings, after being exposed to various treatments under varied lighting conditions for a period of 7 d, were used to examine the expression of three antioxidant genes from the SOD family, namely, copper/zinc SOD (Cu/Zn-SOD), iron SOD (Fe-SOD), and manganese SOD (Mn-SOD). Total RNA was extracted using the RNAprep pure Plant Kit (code: DP432, Tiangen, Beijing, China), and total RNA was transcribed into first-strand cDNA using the HiScript III First-Strand cDNA Synthesis Kit (+gDNA wiper). The first-strand cDNA was used for real-time PCR analysis. The data were analyzed using a real-time quantitative PCR instrument (Applied Biosystems 7500, USA) and the resulting data were calculated using the 2^−ΔΔCT^ method. Primer sequences are shown in the Supplementary Materials (Table S1). The 18SrRNA gene served for the internal control.

### 2.6. Assessment of Mixture Effects

A model based on probability theory was used to compare the theoretically predicted joint response, *JP*_pre_, with the observed joint response, *JR*_obs_, determined during the toxicity testing [19]:

*JR*_pre_ = *R*_M/NPs_ + *R*_GO_ − (*R*_M/NPs_ × *R*_GO_/100) (1)

where *R*_M/NPs_ and *R*_GO_ represent the single effect of M/NPs and GO, respectively. If the *JR*_obs_ was noticeably greater or lower than the *JR*_pre_, the result was adjusted to represent a synergistic or antagonistic effect, respectively. Contrarily, the interaction was only taken into account as an additive effect if there was no discernible difference between the *JR*_obs_ and the *JR*_pre_. 

### 2.7. Data Mining and Statistical Analysis

The fold change (*FC*) of biological response was used to indicate the general level of response variation elicited by the test materials, which was employed when examining each indicator in the seed germination, seedling growth, and intracellular antioxidant activity assays. The *FC* was calculated using the following equation:

(2) $$FC=\frac{avg\;(Sample)}{avg\;(Control)}$$

where avg (Sample) represents the average level (mg/L) of biological response induced by the treated group at each concentration and avg (Control) represents the average level (mg/L) of biological response in the untreated group. Furthermore, the FC was converted into logarithmic form, with 2 serving as the base. The correlations between the changes in biological responses corresponding to each indicator were determined through Pearson correlation coefficient analysis. Furthermore, correlations between the three different SOD family genes and total SOD activity were also ascertained using Pearson correlation coefficient analysis.

By using a one-way analysis of variance with a significance level of *p* < 0.05 (IBM SPSS Statistics for Windows, Ver. 23.0, IBM Corp., Armonk, NY, USA), differences between experimental groups that were statistically significant were identified.

## 3. Results

### 3.1. Physicochemical Characterizations

**Figure 1:**
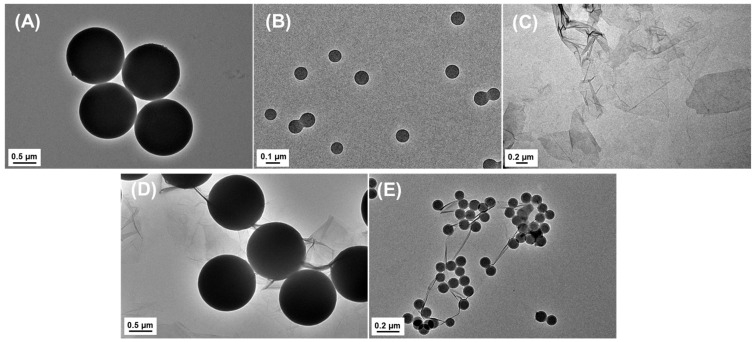
Morphological characterization: (**A**) PSMPs; (**B**) PSNPs; (**C**) GO; (**D**) PSMPs + GO; (**E**) PSNPs + GO via TEM.

Morphologies and structures of PSMPs/PSNPs and GO, both individually and in their combined forms, were characterized through the application of TEM (Figure 1). The TEM images show that both PSMPs (Figure 1A) and PSNPs (Figure 1B) were spheres with smooth surfaces, while GO (Figure 1C) had a lamellar structure unique to graphene-family nanomaterials, characterized by irregular folds and sharp edges decorating its surface, existing in the form of thin sheets. In the mixed systems combining PSMPs/PSNPs with GO (Figure 1D,E), the plastic particles were observed to adhere to the surface of GO. Additionally, the PSNPs were found to encircle GO in clustered formations.

Figure S1 depicts the initial particle size distributions and hydrodynamic diameters of individual and combined PSMPs/PSNPs and GO. As shown in Figure S1A, the particle size distributions of PSMPs and PSNPs, both individually and in conjunction with GO, exhibited a shift towards smaller sizes compared to isolated GO. A slight difference was observed in the particle size distribution between PSMPs/PSNPs used alone and when combined with GO. Additionally, the measurement of hydrodynamic diameters further validated the observed particle size distributions, as presented in Figure S1B.

### 3.2. Seed Germination and Seedling Growth

**Figure 2:**
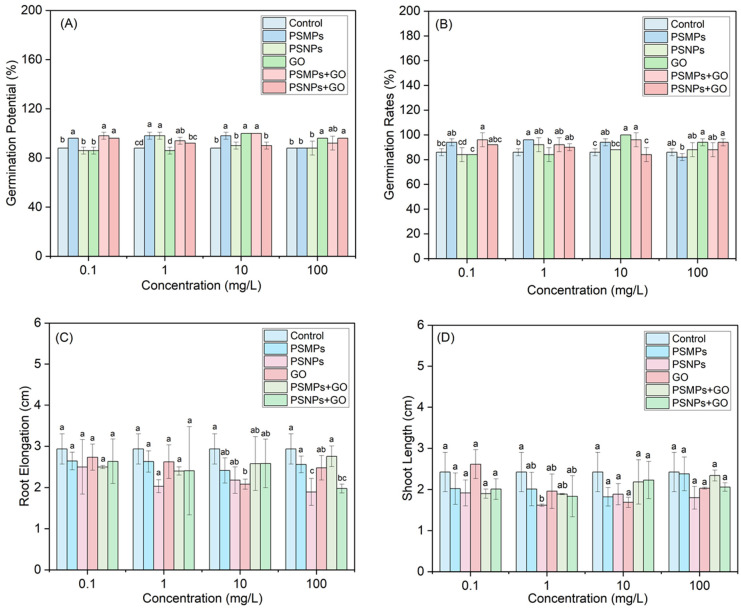
Single and combined effects of PSMPs/PSNPs and GO on seed germination and seedling growth of *Lactuca sativa*: (**A**) germination potential (3 d); (**B**) germination rate (7 d); (**C**) root elongation (7 d); (**D**) shoot length (7 d). Different letters represent statistically significant differences between the exposure treatments within the same concentration (*p* < 0.05).

Single and combined effects of PSMPs/PSNPs and GO on the seed germination and seedling growth of *L. sativa* are presented in Figure 2. Generally, the germination of lettuce seeds treated with different concentrations of PSMPs/PSNPs and GO alone and in combination exhibited either comparable or promoting effects compared to the non-treated control plants (Figure 2A,B). Consequently, it is plausible that the lettuce seeds effectively navigated the challenging conditions encountered during the germination phase, demonstrating well-developed mechanisms to mitigate the impact of stressors. As shown in Figure 2C, there was a significant reduction in the root elongation of lettuce treated with 10 mg/L GO alone, 100 mg/L PSNPs alone, and 100 mg/L PSNPs + 100 mg/L GO mixtures compared to the control group (*p* < 0.05). Additionally, Figure 2D shows that there was a significant reduction in the shoot length of lettuce treated with 1 mg/L PSNPs compared to the control group (*p* < 0.05). Total biomass (fresh weight) analysis showed no significant difference between before and after treatments (Figure S2).

### 3.3. Antioxidant Evaluation in the Seedlings

**Figure 3:**
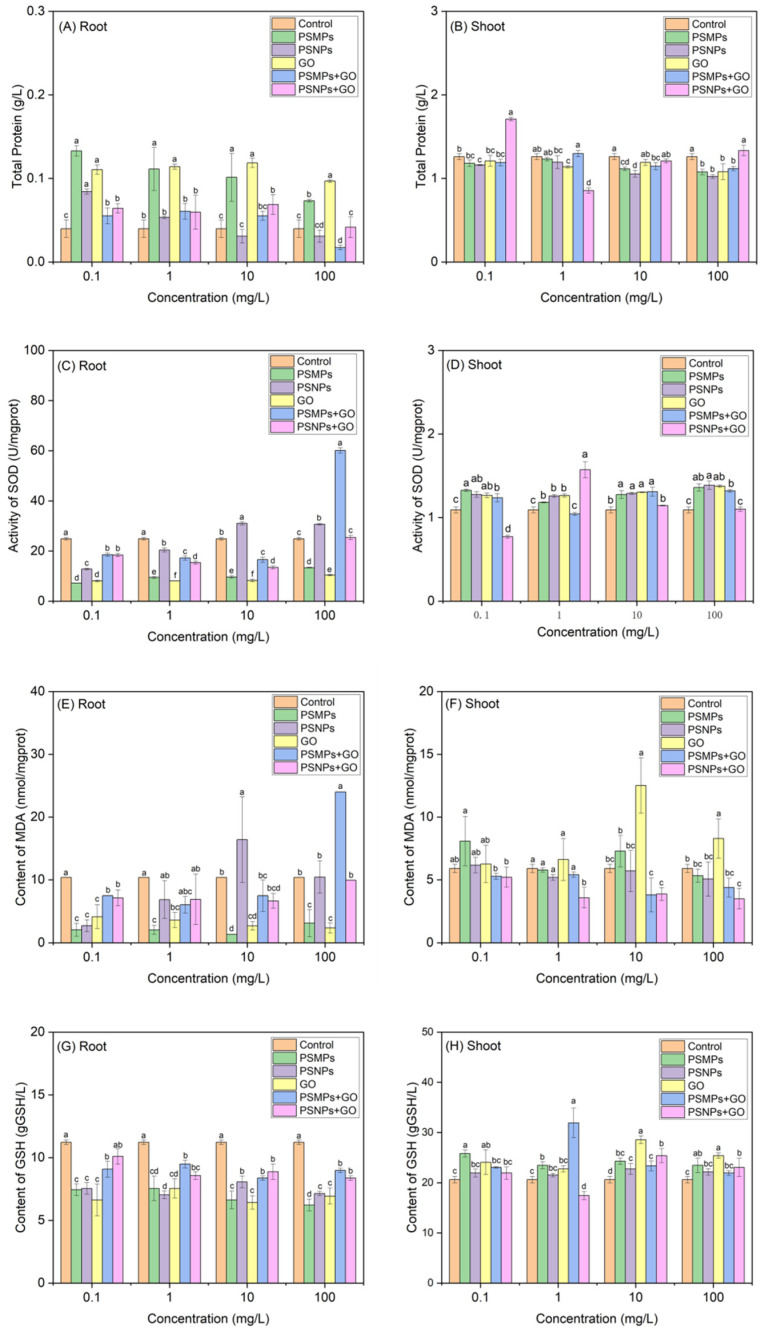
TP content, SOD activity, MDA content, and GSH content in the roots (**A**,**C**,**E**,**G**) and shoots (**B**,**D**,**F**,**H**) of lettuce seedlings (7 d) exposed to single and combined PSMPs/PSNPs and GO. All values are expressed as mean ± standard deviation (*n* = 3). Different letters represent statistically significant differences between the exposure treatments within the same concentration (*p* < 0.05). SOD = superoxide dismutase; MDA = malondialdehyde; GSH = glutathione.

Figure 3 illustrates the impact of single and combined PSMPs/PSNPs and GO on the antioxidant defense system in the roots and shoots of lettuce seedlings. As depicted in Figure 3A, exposure to varying concentrations of PSMPs and GO individually resulted in a significant increase in the TP content of roots compared to the control. For the roots exposed to PSNPs alone, a significant increase in the TP content was observed only at a particle concentration of 0.1 mg/L, relative to the control. Upon exposure to 0.1 mg/L PSMPs + 0.1 mg/L GO mixtures, the TP content of the roots exceeded the control level, whereas the same combination with the mixture concentration of 100 mg/L significantly decreased the TP content of roots. As shown in Figure 3B, exposure to PSMPs (10 and 100 mg/L), PSNPs (0.1, 10, and 100 mg/L), and GO (1 and 100 mg/L) alone treatments significantly reduced the TP content of shoots. Furthermore, the seedlings exposed to PSMPs + GO (10 or 100 mg/L) and PSNPs + GO (1 mg/L) combinations showed a substantial reduction in the TP of shoots, whereas PSNPs + GO (0.1 mg/L) significantly enhanced the TP of shoots compared to the control.

As shown in Figure 3C, when the lettuce seedlings were exposed to varying concentrations of PSMPs and GO alone treatments, the SOD activity in the roots was significantly inhibited. Interestingly, although the SOD activity in the roots was also notably inhibited under single exposure to 0.1 and 1 mg/L of PSNPs, a remarkable enhancement in the SOD activity was observed as the concentration of PSNPs increased to 10 and 100 mg/L. Similarly, the mixtures of PSMPs/PSNPs and GO also inhibited the SOD activity in the roots at the mixed concentrations of 0.1, 1, and 10 mg/L. However, when the mixed concentration increased to 100 mg/L, PSMPs + GO significantly enhanced the SOD activity in the roots, while PSNPs + GO induced the SOD activity to recover to normal levels. As illustrated in Figure 3D, exposure of the shoots to various concentrations of PSMPs, PSNPs, or GO individually resulted in a notable increase in the SOD activity in the shoots. With the exception of the 1 mg/L concentration, PSMPs + GO at all other mixed concentrations consistently resulted in an enhancement of SOD activity in the shoots. Additionally, co-exposure to PSNPs and GO at a mixed concentration of 0.1 mg/L demonstrated an inhibitory effect on SOD activity; however, an increase in the mixed concentration to 1 mg/L led to an increase in SOD activity.

As shown in Figure 3E, the exposure of seedlings to various concentrations of PSMPs and GO treatments individually resulted in a significantly reduced MDA content in the roots compared to the control group. In a similar manner, treatment with 0.1 mg/L PSNPs alone also led to a notable decrease in the MDA content in the roots; conversely, exposure to 10 mg/L PSNPs resulted in a significant increase in the MDA content. Furthermore, co-exposure to a mixed concentration of 0.1 mg/L PSMPs/PSNPs and GO significantly reduced the MDA content in the roots. However, at a mixed concentration of 100 mg/L, the PSMPs + GO mixtures resulted in a significant elevation of MDA levels in the roots. As shown in Figure 3F, the seedlings exposed to either PSMPs or PSNPs in isolation exhibited no significant change in the MDA content in the shoots. However, exposure to 10 and 100 mg/L GO significantly increased the MDA content. Furthermore, co-exposure to various mixed concentrations of PSMPs + GO did not significantly affect the MDA content in the shoots. However, co-exposure to mixed concentrations of 1 and 100 mg/L of PSNPs + GO led to a significant reduction in the MDA content in the shoots.

As shown in Figure 3G, upon individual exposure to PSMPs, PSNPs, and GO, the seedlings exhibited significantly reduced GSH content in the roots compared to the control group. Similarly, when the seedlings were co-exposed to PSMPs and GO at varying mixed concentrations, the GSH content in the roots was also notably decreased. In the case of co-exposure to PSNPs and GO, the GSH content in the roots remained significantly lower than the control group for all mixed concentrations, except for the 0.1 mg/L concentration. As shown in Figure 3H, when the seedlings were exposed to 1 mg/L of PSMPs alone, there was a significant increase in the GSH content in the shoots that was markedly higher than that observed in the control group. Similarly, individual exposure to GO at concentrations of 0.1, 10, and 100 mg/L also resulted in a substantial elevation of GSH levels in the shoots. In experiments involving mixed exposure, where the seedlings were concurrently exposed to PSMPs and GO at the mixed concentrations of 1 and 10 mg/L, the GSH content in the shoots remained significantly elevated compared to the control group. However, at a mixed concentration of 1 mg/L for PSNPs + GO, the GSH content in the shoots exhibited a significant decrease relative to the control. Conversely, as the mixed concentration increased to 10 and 100 mg/L, PSNPs co-exposed with GO led to a significant enhancement in the GSH content in the shoots, exceeding the levels found in the control group.

### 3.4. Expression of SOD Family Genes

**Figure 4:**
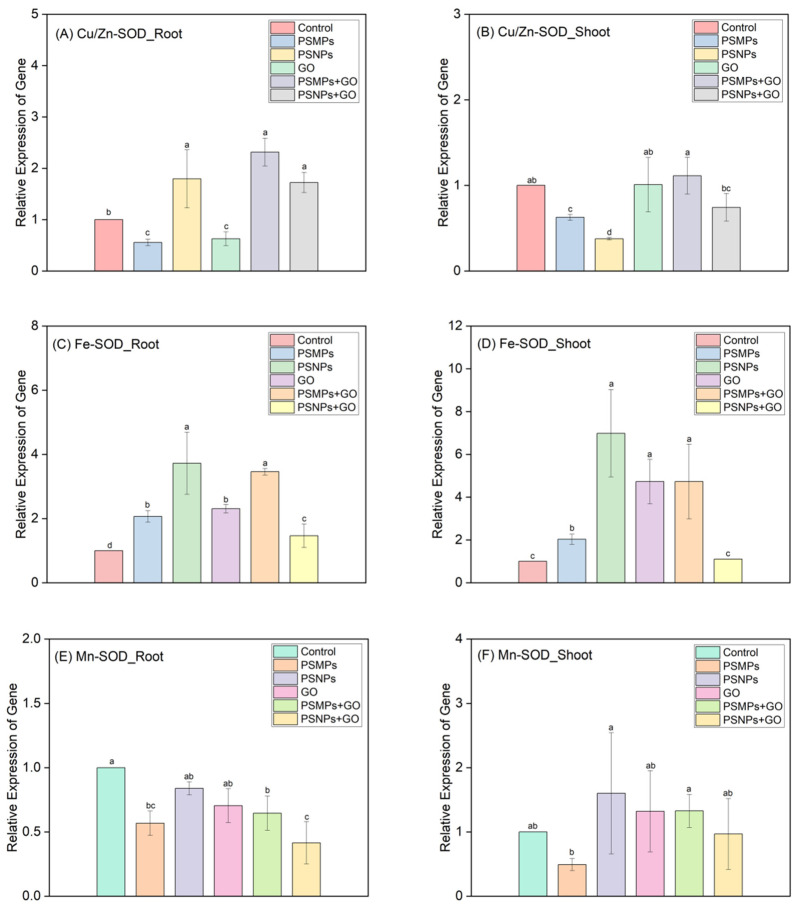
Expression of antioxidant pathway-related genes (Cu/Zn-SOD, Fe-SOD, and Mn-SOD) in the roots (**A**,**C**,**E**) and shoots (**B**,**D**,**F**) of lettuce seedlings (7 d) exposed to single and combined PSMPs/PSNPs and GO at a mixed concentration of 100 mg/L. All values are expressed as mean ± standard deviation (*n* = 3). Different letters represent statistically significant differences between the exposure treatments (*p* < 0.05). SOD = superoxide dismutase.

Figure 4 illustrates the effects of individual and combined exposures to PSMPs/PSNPs and GO on the expression of three SOD family genes in the roots and shoots of lettuce seedlings. Specifically, Figure 4A demonstrates that while PSMPs and GO individually downregulated the expression of Cu/Zn-SOD in the roots, PSNPs alone upregulated it. Notably, when PSMPs or PSNPs were combined with GO, the expression of Cu/Zn-SOD in the roots was significantly upregulated in both cases. Furthermore, Figure 4B reveals that both PSMPs and PSNPs individually downregulated the expression of Cu/Zn-SOD in the shoots, with PSNPs exhibiting a more pronounced effect. In contrast, the exposure to GO alone or in combination with PSMPs/PSNPs did not significantly impact the expression level of this gene in the shoots compared to the control group.

Figure 4C demonstrates that the individual exposure of PSMPs, PSNPs, and GO significantly enhanced the expression of the Fe-SOD gene in the seedling roots, with PSNPs exhibiting a more substantial effect. PSMPs + GO elicited greater upregulation of this gene compared to PSNPs + GO. Additionally, PSMPs + GO resulted in a higher degree of upregulation than when applied individually, whereas PSNPs + GO attenuated the upregulation observed with their individual exposures. Figure 4D reveals that in the seedling shoots, the single treatments with PSMPs, PSNPs, and GO also significantly elevated the expression of the Fe-SOD gene, with PSNPs and GO demonstrating greater efficacy than PSMPs. Upon mixing PSMPs with GO, there was a notable surge in the gene expression, exceeding the level achieved by PSMPs alone and being comparable to that induced by GO alone. Conversely, PSNPs + GO led to a marked decrease in the expression level of this gene compared to their individual exposures, rendering it virtually indistinguishable from those of the control group.

Figure 4E illustrates that both isolated exposure to PSMPs and concurrent exposure with GO significantly diminished the expression of the Mn-SOD gene in the seedling roots. Notably, the inhibitory effect was even more pronounced when PSNPs were combined with GO, leading to a gene expression level that was considerably lower than that observed with either PSNPs or GO alone. Conversely, Figure 4F reveals that isolated exposure to PSMPs significantly downregulated the expression of the Mn-SOD gene in the seedling shoots. However, the effects of other individual or combined treatments on this gene expression in the shoots did not differ significantly from those of the control group.

## 4. Discussion

**Figure 5:**
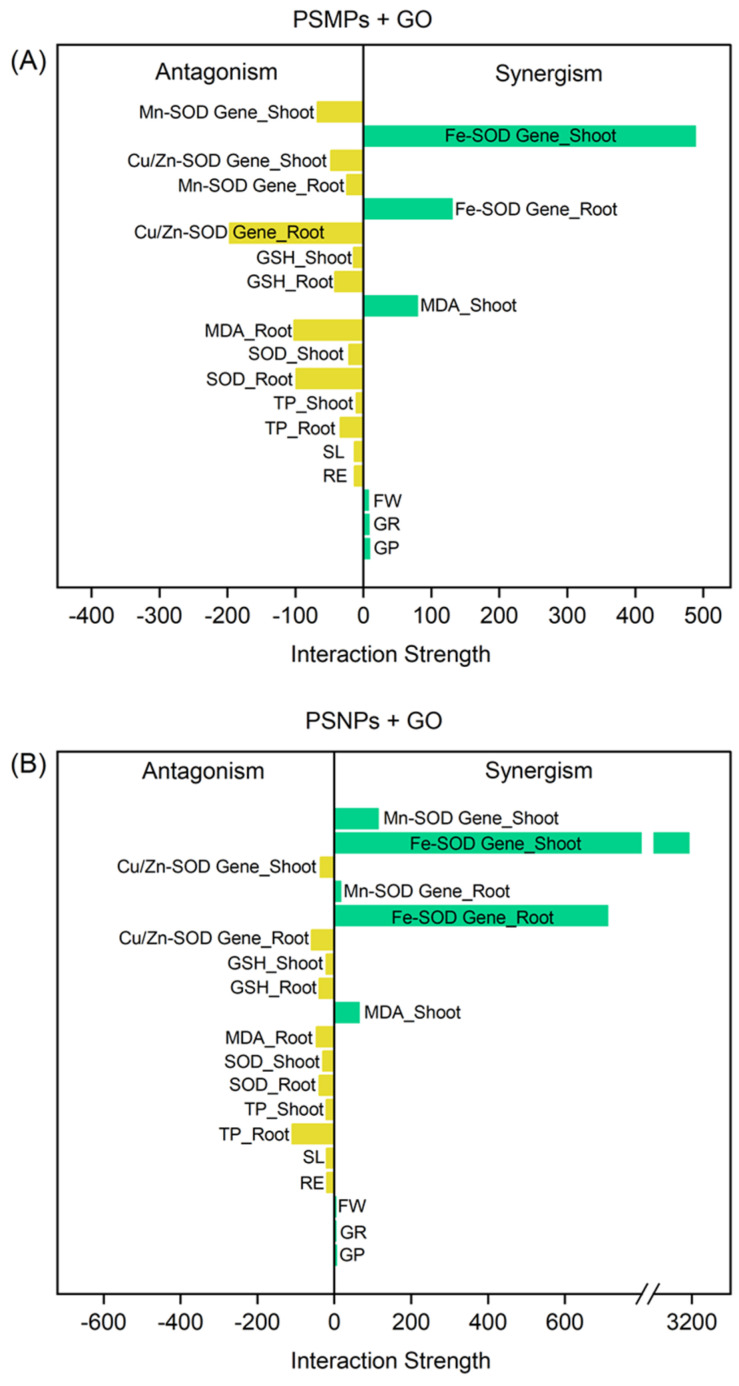
Interaction types between either PSMPs and GO (**A**) or PSNPs and GO (**B**) on lettuce seedling growth parameters, antioxidant activity, and the corresponding gene expression. GP = germination potential; GR = germination rate; FW = fresh weight; RE = root elongation; SL = shoot length; TP = total protein; SOD = superoxide dismutase; MDA = malondialdehyde; GSH = glutathione.

Previous studies have extensively reported on the effects of M/NPs and GO exposure alone on plants [20,21]. However, our study is the first to systematically investigate the effects of mixed exposure to PSMPs/PSNPs and GO of plants, considering growth inhibition at the phenological level, oxidative stress response at the cellular level, and gene expression changes at the molecular level. Figure 5 provides a comprehensive assessment of the combined effects of mixtures of PSMPs/PSNPs and GO on various indicators across different levels of study. At the phenological level, the mixtures of PSMPs/PSNPs and GO exhibited weak synergistic effects on the germination potential, germination rate, and fresh weight of seedlings. However, both combinations produced antagonistic effects on the root elongation and shoot length of seedlings. At the cellular level, co-exposure to PSMPs/PSNPs and GO demonstrated antagonistic effects on TP content, SOD activity, MDA content, and GSH content in tissues. Additionally, there was a synergistic effect on MDA content specifically in shoots. At the molecular level, the mixtures of PSMPs/PSNPs and GO showed antagonistic effects on the expression of Cu/Zn-SOD genes and synergistic effects on Fe-SOD genes in roots and shoots. Furthermore, PSMPs + GO exhibited an antagonistic effect on Mn-SOD gene expression in roots and shoots (Figure 5A), while PSNPs + GO showed a synergistic effect on Mn-SOD genes in the roots and shoots (Figure 5B). Taken together, by analyzing the indicators at each level, we found that the effects of mixed exposure of PSMPs/PSNPs and GO significantly differed from their individual exposure on lettuce seedlings.

Based on analyses of the physicochemical properties mentioned above, it was established that PSMPs and PSNPs can adhere to the surface of GO, and this interaction tends to promote the disagglomeration of GO. On the one hand, the binding of PSMPs and PSNPs to the GO surface may significantly alter the inherent properties of GO, including its charge distribution and hydrophilic/hydrophobic characteristics. These changes, in turn, affect the interaction between GO and plant cells. This alteration is likely to weaken the biological effects of GO and, in some cases, may even exert an antagonistic effect on plants, thereby reducing the potential hazard posed by GO to them. On the other hand, the disagglomeration of GO induced by PSMPs and PSNPs results in the formation of smaller particles. These smaller particles, with their larger specific surface area and higher reactivity, may be more readily absorbed and transported by plant cells, thereby enhancing the toxicity of GO to plants. In this scenario, a synergistic effect may arise, where the combined exposure to PSMPs and PSNPs along with GO causes more severe damage to plants than would occur from exposure to either substance alone.

It is important to recognize that the interaction between PSMPs/PSNPs and GO, along with their combined toxicity to plants, is a complex process influenced by multiple factors. These factors include, but are not limited to, the physicochemical properties of particles, exposure concentrations, exposure durations, types of plant species, growth stages, and physiological conditions of the plants. Consequently, at the phenological, cellular, and molecular levels, combined exposure to PSMPs/PSNPs and GO may result in either synergistic or antagonistic effects, depending on the interplay and dynamic balance of the aforementioned factors. In light of this, future research must urgently explore the interaction mechanisms between M/NPs and GO, as well as their combined effects on plant cells.

**Figure 6:**
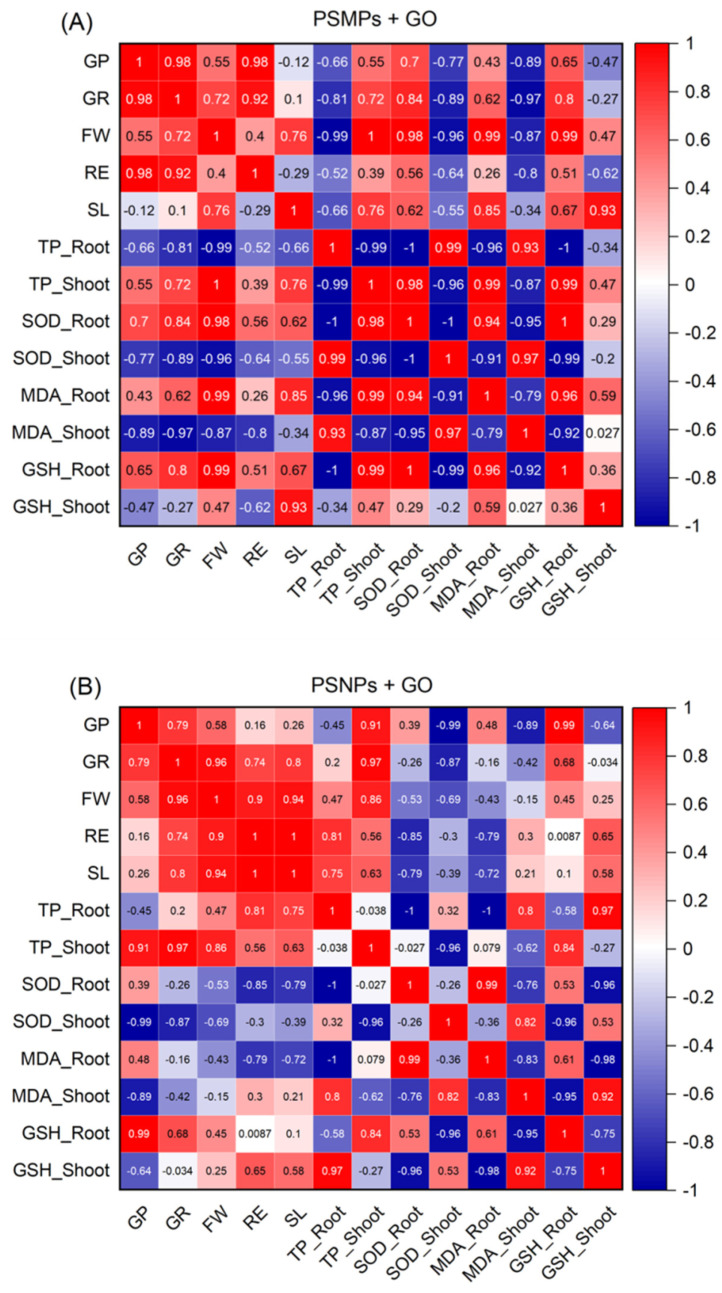
Heat map of correlation coefficients between growth parameters and antioxidant activity in the lettuce seedlings exposed to the PSMPs + GO (**A**) and PSNPs + GO (**B**) combinations. GP = germination potential; GR = germination rate; FW = fresh weight; RE = root elongation; SL = shoot length; TP = total protein; SOD = superoxide dismutase; MDA = malondialdehyde; GSH = glutathione.

Oxidative stress is a typical cellular-level mechanism that affects plant responses to M/NPs and GO [22,23]. Reactive oxygen species (ROS)-modified substances may play integrating roles in the coordination of an organism’s response to stresses [24]. Intracellular concentrations of ROS such as singlet oxygen (^1^O_2_), superoxide (O_2_^−^), hydrogen peroxide (H_2_O_2_), and hydroxyl radical (OH·) are generally very low in normal live organisms [25]. Oxidative stress occurs when there is an imbalance between the generation and scavenging of ROS, leading to ROS accumulation and oxidative damage to plant cells [26]. Antioxidant activity, on the other hand, refers to the range of antioxidant substances and antioxidant enzyme systems present in plants that scavenge ROS and protect cells from oxidative damage [27]. In plants, oxidative stress and antioxidant activity are in an opposing but interdependent state. Oxidative stress is triggered when the production of ROS exceeds the scavenging capacity of the antioxidant system, while the antioxidant system maintains intracellular redox homeostasis and prevents oxidative stress by continuously scavenging ROS. In this study, we established the correlation between the changes in the levels of biomarkers of antioxidant activity and the changes in apparent growth indicators to investigate the mechanism of toxic effects of PSMPs/PSNPs and GO, both alone and in mixed exposure, on different tissues of lettuce seedlings. As can be seen from Figure 6, the data points of each index formed distinct clusters, indicating different correlation profiles compared to the other samples. For either PSMPs + GO (Figure 6A) or PSNPs + GO (Figure 6B), there was a strong correlation between the changes in each apparent growth index and the changes in the levels of biomarkers of antioxidant activity in each tissue fraction. Furthermore, this correlation also differed in the changes of antioxidant chemical activity biomarker levels in different seedling tissues.

**Figure 7:**
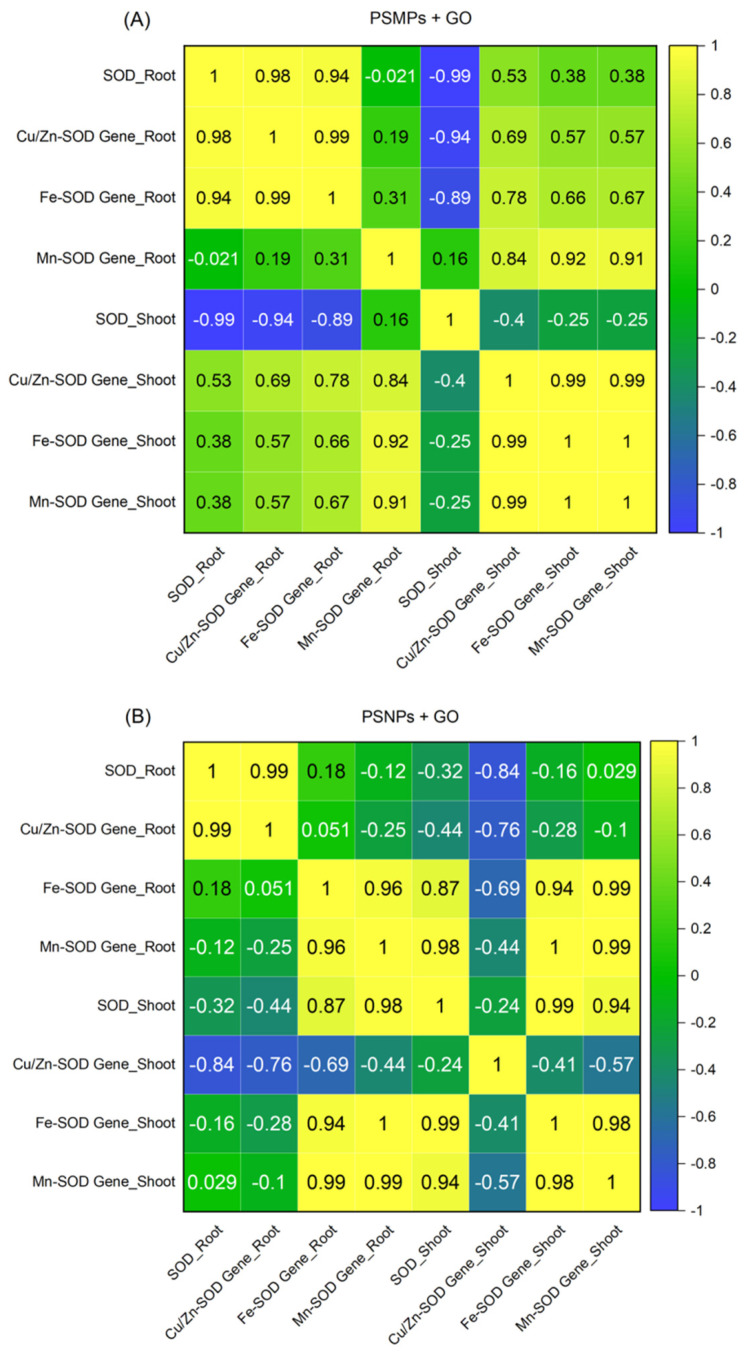
Heat map of correlation coefficients between total superoxide dismutase (SOD) activity and the corresponding SOD family gene expression in the lettuce seedlings exposed to the PSMPs + GO (**A**) and PSNPs + GO (**B**) combinations.

SOD is an important enzyme that serves as the first line of defense in the plant antioxidant system and removes ROS under adverse conditions [28]. The SOD gene family (i.e., Cu/Zn-SOD, Fe-SOD, and Mn-SOD) has been identified in a number of plant species and plays a significant role in plant growth and development [29]. Figure 7 demonstrates the contribution of the three SOD genes to the total SOD activity. As can be seen from Figure 7A, after co-exposure to PSMPs and GO, the three SOD genes in the roots of the seedlings were correlated with the total SOD activity in the following descending order: Cu/Zn-SOD gene > Fe-SOD gene > Mn-SOD gene. Similarly, the three SOD genes in the shoots of the seedlings were correlated with the total SOD activity in the same order: Cu/Zn-SOD gene > Fe-SOD gene > Mn-SOD gene. It can be seen that the Cu/Zn-SOD gene contributed the most to the regulation of oxidative stress effects induced by PSMPs + GO. As shown in Figure 7B, after co-exposure to PSNPs and GO, the three SOD genes in the seedling roots were correlated with total SOD activity in the following descending order: Cu/Zn-SOD gene > Fe-SOD gene > Mn-SOD gene. However, in the seedling shoots, the correlation with total SOD activity was in the following descending order: Fe-SOD gene > Mn-SOD gene > Cu/Zn-SOD gene. This showed that the Cu/Zn-SOD gene contributed the most to the regulation of oxidative stress caused by PSNPs + GO in the roots, whereas the Fe-SOD gene contributed the most to the regulation of oxidative stress caused by PSNPs + GO in the shoots. In addition, the data points of each index formed distinct clusters, indicating different correlation profiles compared to the other samples. Meanwhile, in terms of the regulation of oxidative stress effects by SOD genes, PSMPs + GO exhibited significantly different results from PSNPs + GO. For the PSNPs + GO combination, the contribution of SOD genes was related to the different tissue types of seedlings. Furthermore, the contribution of the three SOD genes to the regulation of total SOD activity in the roots under co-exposure to PSMPs and GO was higher than that of the combination of PSNPs and GO. Additionally, the contribution of the Cu/Zn-SOD gene to the regulation of total SOD activity in the shoots was higher after co-exposure to PSMPs and GO than after co-exposure to PSNPs and GO. However, the regulatory contribution of both the Fe-SOD gene and the Mn-SOD gene to total SOD activity in the shoots after co-exposure to PSNPs and GO was higher than that after co-exposure to PSMPs and GO.

Apart from SOD, other crucial antioxidant indicators, namely, catalase (CAT) and glutathione peroxidase (GPX), may likewise exert significant influence in counteracting stressors [30]. A more thorough evaluation of the antioxidant system, along with its associated antioxidative genes, would offer a refined comprehension of the cellular mechanisms that underlie the observed effects.

To sum up, this study explored the cellular and molecular mechanisms underlying the synergistic effects of PSMPs/PSNPs and GO on the roots and shoots of lettuce seedlings, with a particular focus on oxidative stress and the modulation of the antioxidant defense system mediated by SOD family genes. The results suggest that analogous processes may be present in other plant species. Our findings can serve as a foundational basis for future research examining the effects of both M/NPs and GFNs on a broader spectrum of food crops. Additionally, this contribution may stimulate further inquiry in this domain, thereby enhancing the development of informed policies and practices regarding the utilization and disposal of these micro/nano-particles in agricultural contexts.

## 5. Conclusions

This study confirmed that combined exposure to PSMPs/PSNPs and GO significantly altered lettuce seed germination, seedling growth, antioxidant defense, and related gene expression in the roots and shoots, differing notably from individual exposures, exhibiting either synergistic or antagonistic interactions. The lettuce seeds managed to overcome the stress conditions in the germination stage, having sufficiently well-established mechanisms to counteract the stress factors. Oxidative stress was identified as the primary mechanism of action at the cellular level for the effects of mixed exposure to PSMPs/PSNPs and GO on the seedling roots and shoots. Three SOD family genes were closely associated with the regulation of the antioxidant defense system. Specifically, the Cu/Zn-SOD gene contributed most significantly to the regulation of oxidative stress caused by PSMPs + GO in the roots and shoots. The Cu/Zn-SOD gene also played a major role in regulating oxidative stress caused by PSNPs + GO in the roots, while the Fe-SOD gene was the primary contributor to regulating oxidative stress caused by PSNPs + GO in the shoots. Our findings provide valuable insights for further understanding the cellular and molecular mechanisms underlying the joint effects of M/NPs and GFNs on crop species.

## Data Availability

The original contributions presented in this study are included in the article/Supplementary Materials. Further inquiries can be directed to the corresponding authors.

## Supplementary Materials

The following supporting information can be downloaded at https://www.mdpi.com/article/10.3390/plants13243466/s1. Table S1: Sequences of primer pairs used in the real-time quantitative PCR reactions. All sequences are shown 5′→3′. Figure S1: Intensity-based size distributions by the dynamic light scattering analysis (A) and hydrodynamic diameters (B) of individual and combined PSMPs/PSNPs and GO. All values for the hydrodynamic diameters are expressed as mean ± standard deviation (*n* = 3). Different letters represent statistically significant differences between the exposure treatments (*p* < 0.05). Figure S2: Single and combined effects of PSMPs/PSNPs and GO on the total fresh weight of the seedlings.
