# Supplementary material for: Impacts of Micro/Nanoplastics Combined with Graphene Oxide on Lactuca sativa Seeds: Insights into Seedling Growth, Oxidative Stress, and Antioxidant Gene Expression
Source: Plants (Basel). 2024 Dec 11;13(24):3466. doi: 10.3390/plants13243466 (PMC11679930; doi:10.3390/plants13243466)
Supplement: Supplementary file 1 [file plants-13-03466-s001.zip › plants-3317975-supplementary.pdf]

Supplementary Material

Impacts of Micro/Nanoplastics Combined with Graphene Oxide on *Lactuca sativa* Seeds: Insights into Seedling Growth, Oxidative Stress and Antioxidant Gene Expression

Xuancheng Yuan [1], Fan Zhang [2,*], Zhuang Wang [1,*]

[1] Jiangsu Key Laboratory of Atmospheric Environment Monitoring and Pollution Control, Collaborative Innovation Center of Atmospheric Environment and Equipment Technology, School of Environmental Science and Engineering, Nanjing University of Information Science and Technology, Nanjing 210044, China

[2] College of Environmental Science and Engineering, Yangzhou University, Yangzhou 225127, China

* Corresponding authors:

Fan Zhang, E-mail: f.zhang@yzu.edu.cn; Zhuang Wang, E-mail: zhuang.wang@nuist.edu.cn

4 Pages

2 Figure

1 Table

Table S1. Sequences of primer pairs used in the real-time quantitative PCR reactions. All sequences are shown as 5'→3'.

Primer	Primer sequences	
superoxide dismutase [Cu/Zn]	Forward primer	AATGGCGTACTGCTGGAATCG
superoxide dismutase [Cu/Zn]	Reverse primer	TGGCGAACCTCATCATCTGG
superoxide dismutase [Fe]	Forward primer	GGCTCGTTTGGAGGCCTAAT
superoxide dismutase [Fe]	Reverse primer	ACTCCTCAGGGTCACTTCCA
superoxide dismutase [Mn]	Forward primer	ACGGTGCACACAGATTCCATT
superoxide dismutase [Mn]	Reverse primer	GATTCACATGACCTCCGCCA
18SrRNA	Forward primer	GTGAGTGAAGAAGGGCAATG
18SrRNA	Reverse primer	CACTTTCAACCCGATTCACC

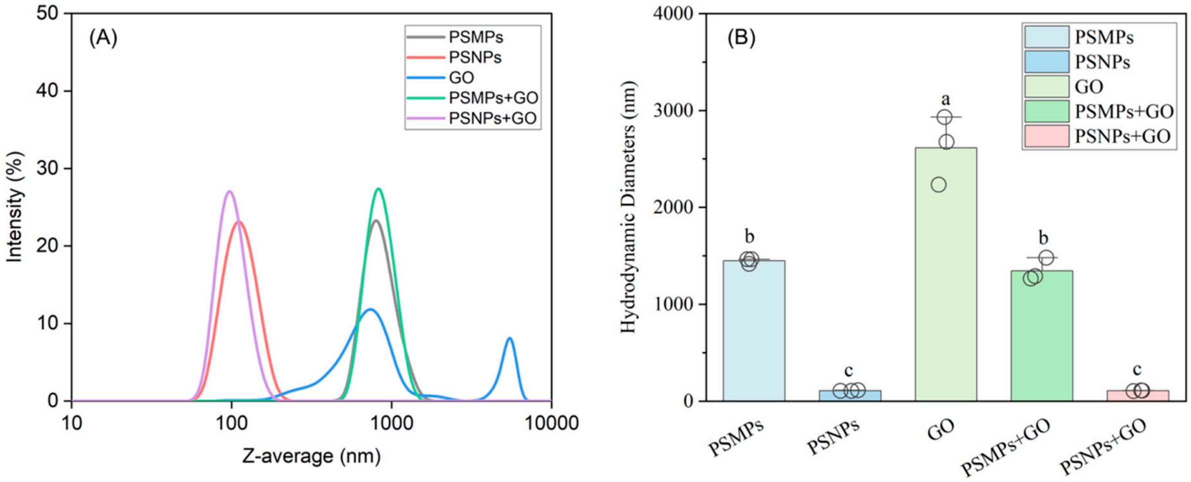

Figure S1. Intensity-based size distributions by the dynamic light scattering analysis (A) and hydrodynamic diameters (B) of individual and combined PSMPs/PSNPs and GO. All values for the hydrodynamic diameters are expressed as mean ± standard deviation (n = 3). Different letters represent statistically significant differences between the exposure treatments ($p < 0.05$).

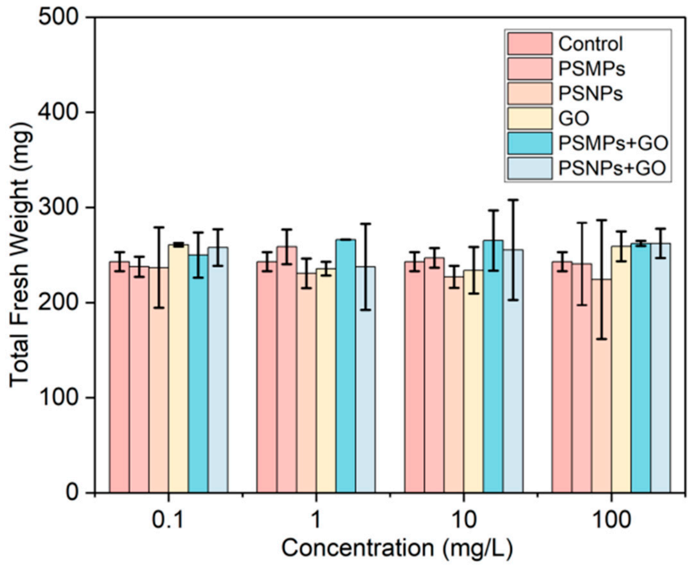

Figure S2. Single and combined effects of PSMPs/PSNPs and GO on the total fresh weight of the seedlings.
